# Enhancing English language acquisition through physical education and motor imagery: assessing enjoyment, English retention, and skill accuracy in adolescent learners

**DOI:** 10.3389/fpsyg.2025.1672418

**Published:** 2025-09-08

**Authors:** Yutong Wu, Xi Dang, Lili Qiao, Ang Li, Hongbo Zhang, Jia He

**Affiliations:** ^1^Geely University of China, Chengdu, China; ^2^Sichuan Normal University, Chengdu, China

**Keywords:** physical exercise, second language learning, skill development, sports education, youth

## Abstract

**Objectives:**

Emerging evidence highlights the benefits of integrating physical activity and cognitive strategies, such as motor imagery (MI), into educational contexts to support language acquisition and skill development. However, the interactive effects of physical education (PE) and MI on second language learning remain underexplored.

**Methods:**

This study investigated the combined impact of PE, MI, and English instruction on vocabulary retention, motor performance, and student enjoyment in junior secondary students. Using a crossover repeated-measures design, 92 students (aged 12–13) participated in seven randomized class sessions incorporating different combinations of PE, MI, and English (alone, in pairs, or all together) over a two-week period. Vocabulary retention was assessed through pre- and post-class English quizzes, motor skill performance was measured using basketball passing accuracy, and enjoyment was evaluated through post-session surveys.

**Results:**

All combinations led to significant post-session improvements in English quiz scores (*p* < 0.001), with the highest improvements observed in the PE + MI + English condition. Similar patterns were found for passing accuracy, where PE-containing sessions produced the greatest improvements, particularly when combined with MI and English (*p* < 0.001). Enjoyment scores were also significantly higher in integrated conditions, especially PE + MI + English, suggesting increased engagement. A significant interaction between class type and time was found for both vocabulary and motor skill performance (*p* < 0.001), with very large effect sizes (ηp^2^ = 0.772 and 0.699, respectively).

**Conclusion:**

These findings suggest that integrating physical, cognitive, and linguistic activities may positively impact both learning outcomes and student experience, supporting a multidisciplinary, multimodal approach to education. However, longitudinal studies are needed to identify potential long-term effects on consolidated learning.

## 1 Introduction

Recent advances in cognitive science and educational psychology suggest the benefits of integrating physical movement with language learning, grounded in the framework of embodied cognition (Shapiro, 2007). According to embodied cognition theory (Leitan and Chaffey, 2014), language and thought are not isolated mental processes but are fundamentally shaped by bodily actions and sensorimotor experiences. In this view, learning a second language becomes more effective when it is tied to physical context—particularly actions that can be seen, felt, or imagined (Atkinson, 2010). Within Physical Education (PE), learners engage in concrete motor tasks that offer natural opportunities to contextualize English vocabulary and grammatical structures, especially action verbs, prepositions, and instructional phrases (Forey and Cheung, 2019). These physically grounded interactions enhance retention by activating dual coding mechanisms, where verbal and non-verbal information is processed in parallel (Mir et al., 2023), supporting stronger memory encoding and recall. By engaging both channels, learners form richer mental representations that are more resistant to forgetting. This dual-channel processing is a core principle of multimodal learning (Paivio, 1986), helping explain why pairing movement with language input can yield stronger memory encoding and retrieval in second language acquisition.

Motor imagery (MI) aims to serve as a cognitive bridge between movement and language by mentally simulating physical actions, engaging brain areas involved in both motor planning and verbal processing (Munzert et al., 2009). Research suggests that MI may activate neural networks similar to those involved in actual movement (Sharma and Baron, 2013), which can reinforce physical skill acquisition (Ingram et al., 2016) and, when guided through language, deepen comprehension of action-related vocabular (Bayram et al., 2023). Thus, it is hypothesized that embedding MI within PE sessions conducted in English may enable learners to rehearse both motor sequences and linguistic structures simultaneously, increasing cognitive engagement.

The available research suggests that integrating PE with English language learning can be mutually beneficial. PE-in-CLIL (Content and Language Integrated Learning) approaches have shown significant improvements in students’ oral communication skills (Coral and Lleixà, 2016). Physical activity may favour cognitive functions and memory, leading to better academic performance, including in English (Yurko et al., 2019). Integrated PE and English lessons can increase motivation for language learning and promote a healthy lifestyle (Bashta and Zalutskaya, 2021). Combining PE theory with practical sessions cultivates collaboration skills and multi-faceted thinking abilities (Yamaki and Iwata, 2020). These integrated approaches not only improve physical skills but also develop critical thinking and create interconnections between disciplines (Yamaki and Iwata, 2020).

MI activates similar neural substrates as motor execution, potentially facilitating motor skill learning (Anwar et al., 2011) and improving language performance (Bayram et al., 2023). Studies have shown that kinesthetic motor-imagery training can lead to better performance in lexical-semantic access tasks compared to static visual imagery training (Bonnet et al., 2022) This improvement in language comprehension can be attributed to the relationship between the language and motor systems. Additionally, technology-based interventions incorporating L2 imagery techniques have showed benefits for learner engagement and language acquisition in English for Academic Purposes courses (Gonzalez Mujico and Lasagabaster, 2019). Although there are some positive preliminary results, research focusing on MI and English acquisition is still very limited, so further studies are needed to fully understand the potential impact.

Although existing evidence points to potentially beneficial effects of PE classes on language acquisition and the use of MI for both motor skill and language learning, findings in this area remain inconsistent. Some studies report positive outcomes of MI for vocabulary retention and skill transfer (Bonnet et al., 2022), yet research replicating and confirming these results is scarce. This scarcity may contribute to discrepancies in findings and practical applications, potentially arising from differences in research approaches, small sample sizes, short intervention durations, and a lack of well-controlled comparisons between instructional combinations (e.g., directly comparing MI with PE). Moreover, no study has examined the interaction between PE and MI in relation to second language acquisition—particularly in English—while systematically addressing these methodological limitations. Combining these interventions may also enhance student engagement through increased enjoyment during classes, yet this potential remains underexplored in integrated designs. This gap underscores the originality of the present study, which adopts a crossover repeated-measures design to investigate the combined and separate effects of PE, MI, and English instruction. Specifically, it examines how different configurations of these elements (individually, in pairs, or all together) influence post-class vocabulary retention, student enjoyment, and skill accuracy, thereby contributing new insights toward the development of engaging, multidisciplinary approaches to skill development.

## 2 Methods

### 2.1 Study design

**Figure 1:**
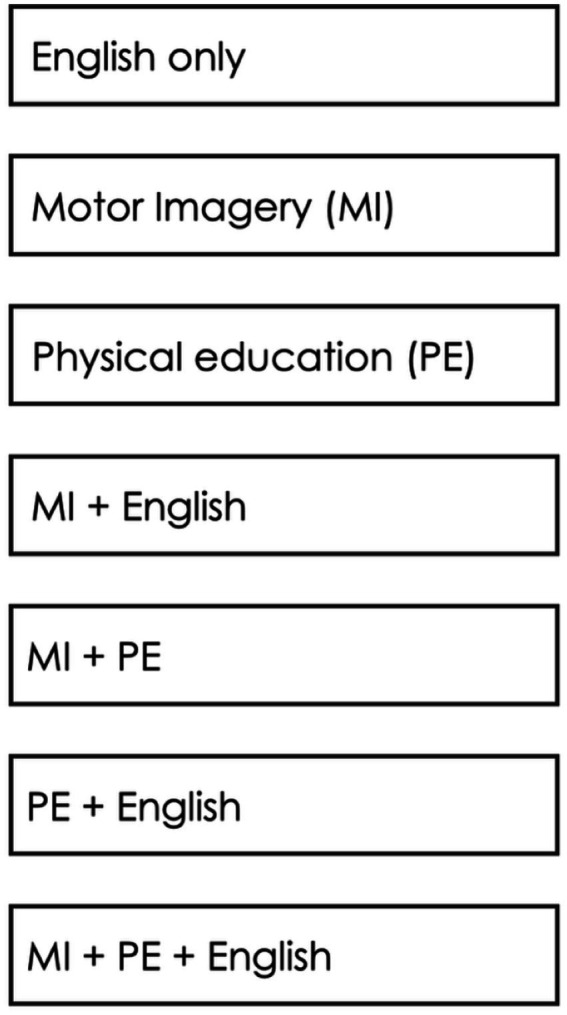
Different class combinations students experienced during 2 weeks. PE, physical education; MI, motor imagery.

This study employed a crossover repeated-measures design, in which the same group of students participated in seven randomized class sessions (Figure 1). This design was chosen over a between-subjects approach to control for inter-individual variability in factors such as baseline motor skills, cognitive ability, and language proficiency. By having each student serve as their own control, this design minimizes confounding effects of individual differences. Furthermore, the repeated-measures nature of the design allows for a direct comparison of the impact of each instructional condition within the same participants, providing a more accurate evaluation of the effects of PE, MI, and English instruction alone and in combination.

The sessions integrated PE, MI, and English instruction in various combinations—delivered individually, in pairs, or all three together. The sessions took place over two consecutive weeks, during which students attended three PE classes per week. Figure 1 illustrates the different class combinations students experienced during this period. Each student participated in one session of each combination, with measurements taken both before and after each class. To minimize sequencing bias, the order and sequence of conditions were randomized. The procedure, detailed later in the section on classes, involved implementing three different condition orders and assigning students classes randomly to the sequences using opaque envelopes. Given the educational context, the randomization was carried out within the three classes of student participants. The tests were administered under similar conditions before and after each session, using reliable measures.

### 2.2 Participants

To determine the *a priori* sample size required for two-way repeated measures ANOVA, a power analysis was conducted. Based on the aim of recruiting approximately 90 participants, the effect size was targeted to achieve this sample size. Specifically, assuming an f^2^ of approximately 0.028, which falls within the range typically considered a small effect size according to Cohen’s conventions. The desired statistical power was set at 0.80, and the alpha level (Type I error rate) was established at 0.05. Assuming a moderate correlation of 0.5 among the repeated measures, and utilizing specialized statistical software (e.g., G*Power 3.1), the estimated minimum total sample size required to detect the hypothesized interaction effect with the specified power and alpha level is approximately 90 participants.

Convenience sampling was conducted at a junior secondary school, involving male and female students aged 12 to 13. The school had implemented various pedagogical and didactic innovations, including a multidisciplinary approach that integrated PE and English. This integration was part of the school’s strategy to enhance teaching effectiveness and support second language learning. As part of this initiative, the PE and English departments collaboratively developed a combined teaching approach. This study followed that initiative, analyzing the effects of the integrated classes through a pre- and post-intervention design, focusing on the interactions between the two disciplines.

Among the students, 92 (46 boys and 46 girls) were identified as volunteers based on the following eligibility criteria: (i) participation in all classes throughout the observation period; (ii) ability to attend both pre- and post-class evaluations; and (iii) being between 12 and 13 years old. Participants were excluded from the analysis if they (i) missed any classes during the period or (ii) declined to participate in the evaluations. At baseline, before the study began, the students were assessed using the Cambridge English: Preliminary for Schools (PET) test. Fifteen students demonstrated a B1 level (Intermediate), 49 an A2 level (Elementary), and 27 an A1 level (Breakthrough).

The students and their parents were informed about the study and the evaluation process. They were told that although the classes were part of the regular curriculum, participation in the pre- and post-evaluations was voluntary. It was made clear that there would be no obligation to take part in the assessments and that non-participation would not result in any penalties. All students and their parents agreed to participate, and the parents signed an informed consent form. Furthermore, the study design was approved by the ethics committee of Sichuan Normal University (approval code: 2025LS0058), in accordance with ethical standards for research involving youth populations.

### 2.3 The classes

The classes were conducted according to the regular school schedule and formed part of the students’ standard curriculum. To minimize sequencing effects, students experienced the activities in different orders. Given the large number of possible permutations (7 conditions – 5,040 possible sequences) and the practical constraints of implementing all sequences in a school setting, only three specific combinations were used, which were selected by generating random sequences from the multiple possible options. These were: Ordering 1 (PE → MI → ENG → PE + MI → PE + ENG → MI + ENG → PE + MI + ENG), Ordering 2 (ENG → PE + MI + ENG → PE + MI → MI → MI + ENG → PE + ENG → PE), and Ordering 3 (MI + ENG → PE → PE + MI → PE + MI + ENG → PE +  ENG → MI → ENG).

Each class was co-taught by an English teacher and a PE teacher, both certified professionals with over five years of experience. The same teachers and teaching methods were used throughout to ensure consistent conditions for all students. The MI and PE classes were conducted in both Mandarin (for general instructions) and English (for specific communication in the sports contexts and interaction with peers). Students were required to communicate in English to describe their actions and to interact with peers and teachers during PE activities. To unify the content across the three main areas—English, MI, and PE—basketball was chosen as the central theme, specifically focusing on passing and match-passing scenarios, a topic of high interest among the students. This thematic approach provided a core foundation across the different sessions. Table 1 shows the main content and pedagogical strategies used in the sessions.

**Table 1:** Description of the core contents and pedagogical approaches for each session.

Class	Main content	Pedagogical approaches
English only	Vocabulary: pass, teammate, ball, behind, left	Language games and role-play
MI only	Visualize passing to teammate, choosing direction	Mental rehearsal, internal focus
PE only	Execute passes in drills and 3v3 games	Physical execution of skill
MI + English	Visualize passing while listening to English narration	Combined mental + linguistic
MI + PE	Visualize then immediately practice passing	Motor priming via MI
PE + English	Perform passing drills using English commands	Physical execution + contextual language use
MI + PE + English	Full integration: visualize + perform + verbalize passing	Maximally embodied, contextual learning

The specific details of the English classes are presented in Table 2, which summarizes the schedule, activities, and their descriptions. All themes were specifically designed around the context of basketball, with a focus on key vocabulary related to the sport and useful phrases to support communication with peers.

**Table 2:** Description of the activities and contents for the English session.

Time	Activity	Description
0:00–0:05	Warm-up game: “Simon Says” focused on basketball	Instructor uses only English: “Pass the ball,” “Stand behind your teammate.” Students follow commands with gestures or classroom props (paper balls).
0:05–0:15	Vocabulary presentation	Show flashcards: “pass,” “ball,” “teammate,” “left,” “right,” “behind.” Pronounce, repeat, and do gestures.
0:15–0:25	Sentence practice in Pairs	Each pair uses puppets/toys or drawings to act out: “I pass the ball,” “You are behind me,” etc.
0:25–0:35	Role play game: “coach and player”	One student gives instructions in English (e.g., “Pass left!”), the other moves small figures on a paper court.
0:35–0:40	Group sentence chain	Students sit in a circle: one says “I pass to ___,” the next continues.
0:40–0:60	Quick recap + enjoyment rating	Review vocabulary; students complete enjoyment scale and do post-test English quiz, as well as, technical accuracy.

Table 3 presents the activities and content covered in the MI classes. These sessions built on the students’ previous familiarity with the MI approach, extended through earlier learning experiences. In this specific context, the focus was on integrating basketball-related content into their imagery, aiming to enhance the overall learning process.

**Table 3:** Description of the activities and contents for the motor imagery.

Time	Activity	Description
0:00–0:05	Intro to MI (Mandarin)	Explain the context of MI and the tasks
0:05–0:10	Body relaxation	Eyes closed, deep breaths, muscle relaxation to enhance focus.
0:10–0:25	Guided imagery	The teacher describes the full scenario as “You see your teammate on the right… You bounce-pass the ball. They run left.” using vivid sensory cues as sound of shoes or feel of ball.
0:25–0:35	Individual imagery task	Students pick one pass type (chest or bounce), close eyes, and imagine passing in a 3v3 game context.
0:35–0:40	Draw or write scene	Students sketch or write (in Mandarin) what they imagined happening in the game
0:40–0:60	Quick Recap + enjoyment rating	Review vocabulary; students complete enjoyment scale and do post-test English quiz, as well as, technical accuracy.

Table 4 presents the activities and content covered in the PE classes. The sessions focused on the passing skill, approached through a variety of exercises—some aimed specifically at developing the skill itself, and others incorporating it into game-like contexts (i.e., mini 3v3). The main instruction was delivered in Mandarin.

**Table 4:** Description of the activities and contents for the motor imagery.

Time	Activity	Description
0:00–0:05	Dynamic warm-up	Jogging, high knees, shoulder circles. Ball passing in circle to activate readiness.
0:05–0:20	Passing drills	(1) Partner chest passes (10 reps), (2) Bounce passes, (3) Passing while moving.
0:20–0:30	Passing under pressure drill	3v1 keep-away game. One defender tries to intercept while three teammates pass.
0:30–0:40	Mini 3v3 game	Passes only count if done within 3 s.
0:40–0:60	Quick recap + enjoyment rating	Review vocabulary; students complete enjoyment scale and do post-test English quiz, as well as, technical accuracy.

Table 5 presents the activities and content covered in the combined English and MI classes. These sessions integrated activities from both subjects, with a specific emphasis on using English as the primary language for both instruction and student communication. This was especially required when referring to key basketball terms and contexts.

**Table 5:** Description of the activities and contents for the English + motor imagery.

Time	Activity	Description
0:00–0:05	Warm-up game: “Simon Says” focused on basketball	Instructor uses only English: “Pass the ball,” “Stand behind your teammate.” Students follow commands with gestures or classroom props (paper balls).
0:05–0:15	Vocabulary presentation	Show flashcards: “pass,” “ball,” “teammate,” “left,” “right,” “behind.” Pronounce, repeat, and do gestures.
0:15–0:25	Sentence practice in Pairs	Each pair uses puppets/toys or drawings to act out: “I pass the ball,” “You are behind me,” etc.
0:25–0:30	Body relaxation	Eyes closed, deep breaths, muscle relaxation to enhance focus.
0:30–0:40	Guided imagery	The teacher describes the full scenario as “You see your teammate on the right… You bounce-pass the ball. They run left.” using vivid sensory cues as sound of shoes or feel of ball.
0:40–0:60	Quick recap + Enjoyment rating	Review vocabulary; students complete enjoyment scale and do post-test English quiz, as well as, technical accuracy.

Table 6 outlines the activities and content addressed in the integrated English and PE sessions. These classes combined elements from both subjects, with a strong emphasis on using English as the main language for instruction and student interaction—particularly during physical tasks that involved calling for the ball or performing specific movements.

**Table 6:** Description of the activities and contents for the English + physical education.

Time	Activity	Description
0:00–0:05	Warm-up game: “Simon says” focused on basketball	Instructor uses only English: “Pass the ball,” “Stand behind your teammate.” Students follow commands with gestures or classroom props (paper balls).
0:05–0:15	Vocabulary presentation	Show flashcards: “pass,” “ball,” “teammate,” “left,” “right,” “behind.” Pronounce, repeat, and do gestures.
0:15–0:25	Sentence practice in Pairs	Each pair uses puppets/toys or drawings to act out: “I pass the ball,” “You are behind me,” etc.
0:25–0:30	Passing drills	(1) Partner chest passes (10 reps), (2) Bounce passes, (3) Passing while moving.
0:30–0:40	Passing under pressure drill	3v1 keep-away game. One defender tries to intercept while three teammates pass.
0:40–0:60	Quick recap + enjoyment rating	Review vocabulary; students complete enjoyment scale and do post-test English quiz, as well as, technical accuracy.

Table 7 presents the activities and contexts explored in the combined MI and PE classes. Each session focused specifically on passing skills, with Mandarin used as the language of instruction.

**Table 7:** Description of the activities and contents for the motor imagery + physical education.

Time	Activity	Description
0:00–0:05	Intro to MI (Mandarin)	Explain the context of MI and the tasks
0:05–0:10	Body relaxation	Eyes closed, deep breaths, muscle relaxation to enhance focus.
0:10–0:25	Guided imagery	The teacher describes the full scenario as “You see your teammate on the right… You bounce-pass the ball. They run left.” using vivid sensory cues as sound of shoes or feel of ball.
0:25–0:30	Passing drills	(1) Partner chest passes (10 reps), (2) Bounce passes, (3) Passing while moving.
0:30–0:40	Passing under pressure drill	3v1 keep-away game. One defender tries to intercept while three teammates pass.
0:40–0:60	Quick recap + enjoyment rating	Review vocabulary; students complete enjoyment scale and do post-test English quiz, as well as, technical accuracy.

Table 8 provides a detailed description of the session that integrated content and activities from English, MI, and PE. In this case, the majority of the session was conducted in English, requiring students to express themselves and interact in English to the best of their ability—consistent with all sessions where English was combined with MI and PE.

**Table 8:** Description of the activities and contents for the motor imagery + physical education.

Time	Activity	Description
0:00–0:05	Warm-up game: “Simon says” focused on basketball	Instructor uses only English: “Pass the ball,” “Stand behind your teammate.” Students follow commands with gestures or classroom props (paper balls).
0:05–0:10	Body relaxation	Eyes closed, deep breaths, muscle relaxation to enhance focus.
0:10–0:25	Guided imagery	The teacher describes the full scenario as “You see your teammate on the right… You bounce-pass the ball. They run left.” using vivid sensory cues as sound of shoes or feel of ball.
0:25–0:30	Passing drills	(1) Partner chest passes (10 reps), (2) Bounce passes, (3) Passing while moving.
0:30–0:40	Passing under pressure drill	3v1 keep-away game. One defender tries to intercept while three teammates pass.
0:40–0:60	Quick recap + enjoyment rating	Review vocabulary; students complete enjoyment scale and do post-test English quiz, as well as, technical accuracy.

### 2.4 Measurements

All students participating in the study were tested both before and after the class using an English quiz and a passing accuracy assessment. Additionally, after the class, participants completed an enjoyment questionnaire related to the content they experienced. These evaluations were administered by members of the research team who did not take part in teaching the classes, in order to minimize potential evaluation bias.

### 2.5 English quiz

A topic-specific questionnaire was developed by the research team based on the students’ proficiency levels and the class content. After the initial draft, three independent experts reviewed the questionnaire blindly and provided feedback. The researchers then revised the questionnaire accordingly. Following this, a brief pilot test was conducted with 10 students who were not part of the main study to ensure the test was appropriate for their level and relevant to the learning context.

Once finalized, the quiz was administered both before and after the classes. Table 9 presents an example of the English quiz; although minor adjustments were made from class to class, all students received quizzes that followed the same structure and all students received the same quizzes over the time. These adjustments involved only vocabulary and terminology, while the overall format and components remained consistent. The quiz included sections on vocabulary, reading comprehension, writing, and listening. All tests were scored and reviewed by the same researchers, who were blinded to the specific classes the students attended. The final score was the total number of points earned on the quiz, ranging from 0 to a maximum of 30 points.

**Table 9:** Example of the English quiz.

Examples of questions introduced
Part A: multiple choice vocabulary (6 points)
What does “pass” mean in basketball? (a) To run quickly; (b) To give the ball to a teammate; (c) To shoot the ball
What is a “dribble”? (a) Throwing the ball; (b) Bouncing the ball while moving; (c) Catching the ball
If a player “shoots” the ball, what do they do? (a) Throw the ball into the basket; (b) Pass the ball to a friend; (c) Stop the game
Part B: sentence completion (6 points)
Fill in the blanks with the correct word from the box: (pass, dribble, shoot, team, basket)
I _______ the ball to my friend.
To score points, you must put the ball in the _______.
When you move with the ball, you _______ it on the floor.
Part C: reading comprehension (6 points)
Read the passage and answer the questions. “In basketball, players work together as a team. One player passes the ball to another. Then, a player may dribble the ball to move closer to the basket. When the player is ready, they shoot the ball to try to score points.” (a) How do players move the ball to a teammate?; (b) What do players do to move closer to the basket?; (c) Why do players shoot the ball?
Part D: short writing (6 points)
Write 2–3 sentences about what you learned about basketball today using these words: pass, dribble, shoot, basket, team
Part E: listening task (6 points)
Audio narrative: “Today, Tom and his friends are playing basketball. Mike passes the ball to Tom. Tom catches the ball and starts to dribble. He moves quickly toward the basket. Then, Tom shoots the ball. The ball goes into the basket, and Tom scores a point! Everyone on the team is happy.”
Listen to the audio about a basketball game. Then answer the questions: Who passes the ball to Tom? (a) Mike; (b) Lisa; (c) John
What does Tom do after he gets the ball? (a) Dribbles; (b) Shoots; (c) Runs away
Did Tom score a point? (a) Yes; (b) No

### 2.6 Passing skill accuracy

To assess basketball players’ passing precision and side-to-side agility, a 30-s drill involving passing against a wall was conducted (Wei et al., 2025). During the test, participants stood behind a marked line set 2.45 meters from the wall. The wall featured six square targets, each measuring 60 by 60 centimeters, arranged alternately at two different heights: some targets were placed so their bottom edges were 1.5 meters off the ground, while others were positioned at 90 centimeters. Players executed chest passes following a specific order, moving laterally to retrieve the ball while staying behind the designated line. Scoring awarded 2 points for passes hitting any part of a target square or its edges, and 1 point for passes that landed on the wall in the spaces between targets. The sum of these points during the 30-s interval served as a measure of each player’s passing accuracy and skill.

### 2.7 Short form of foreign language enjoyment scale

The short form of the Foreign Language Enjoyment Scale (S-FLES) was used in this study. This scale has been validated and tested for reliability, and is considered a simpler tool for examining individual differences in language learning (Botes et al., 2021). The questionnaire consists of 9 items: 1. The teacher is encouraging, 2. The teacher is friendly, 3. The teacher is supportive, 4. I enjoy it, 5. I’ve learned interesting things, 6. I am proud of my accomplishments, 7. We form a tight group, 8. We laugh a lot, and 9. We share common “legends.” Items 1 to 3 comprise the Teacher Appreciation subscale, items 4 to 6 form the Personal Enjoyment subscale, and items 7 to 9 make up the Social Enjoyment subscale. Each item is rated on a 5-point Likert scale, with 1 meaning “strongly disagree” and 5 meaning “strongly agree.” The scale was translated into Mandarin with the support of three experts to ensure accuracy of terminology. Additionally, the scale was introduced to students prior to the study period to familiarize them with the questions and improve the accuracy of their responses. The questionnaires were administered individually, and researchers remained available nearby to clarify any doubts about the interpretation of items. The final scores represented the sum of the individual scores given by the students.

### 2.8 Statistical analysis

Statistical analysis was conducted using a two-way repeated measures ANOVA to examine the effects of type of class and pre-post assessment on the English quiz and passing accuracy variables. This approach allowed us to assess both effect of the class (pre-post) and analyze variations on the class type effect, as well as their interaction. Effect sizes were calculated using partial eta squared (η^2^) to determine the magnitude of significant effects, with values interpreted according to conventional benchmarks (small: 0.01, medium: 0.06, large: 0.14). When significant interactions or main effects were found, *post hoc* pairwise comparisons with Bonferroni correction were performed to identify specific group differences. For enjoyment scale where only post-session data was available (no pre-post comparison), a one-way ANOVA was conducted to compare the class types at that single time point. To examine the potential effects of sequencing order, a one-way ANOVA was conducted on the mean differences (post–pre) in quiz scores, passing rates, and enjoyment ratings. For pairwise comparisons, Bonferroni corrections were applied. All analyses were conducted using SPSS (version 27), with a significance level set at *p* < 0.05.

## 3 Results

Table 10 presents the English quiz scores before and after the classes, along with the post-class passing accuracy rates and enjoyment levels. A significant two-way interaction between class and time, *F* = 308.785, *p* < 0.001,ηp2 = 0.772, was observed for the English quiz scores. There was also a significant two-way interaction between condition and time, *F* = 210.933, *p* < 0.001, ηp2 = 0.699 for the passing accuracy. There was also a significant main effect of class on enjoyment scores, *F* = 4223.896, *p* < 0.001, ηp2 = 0.979.

**Table 10:** Means and standard deviations for the English quiz scores, basketball passing accuracy, and reported enjoyment after the classes.

Class	Quiz pre	Quiz post	Quiz (post-pre)% difference	Quiz post-pre (p-value)	Passing accuracy pre	Passing accuracy post	Passing (post-pre) % difference	Passing post-pre (p-value)	Enjoyment post
English (ENG)	13.05 ± 2.57	17.35 ± 2.66	32.95	*p* < 0.001	48.40 ± 6.12	48.21 ± 6.09	−0.39	*p* = 0.371	27.41 ± 0.89
Motor Imagery (MI)	13.22 ± 2.66	13.86 ± 2.63	4.84	*p* < 0.001	48.02 ± 6.14	53.29 ± 6.80	10.97	*p* < 0.001	22.38 ± 0.81
Physical Education (PE)	13.21 ± 2.46	13.70 ± 2.58	3.71	*p* < 0.001	48.07 ± 6.14	60.67 ± 9.19	26.21	*p* < 0.001	37.43 ± 0.88
ENG + MI	13.09 ± 2.33	17.84 ± 2.87	36.29	*p* < 0.001	48.21 ± 5.97	54.53 ± 6.13	13.11	*p* < 0.001	30.53 ± 0.98
MI + PE	13.36 ± 2.74	13.82 ± 2.67	3.44	*p* < 0.001	48.14 ± 6.19	64.43 ± 8.98	33.84	*p* < 0.001	38.40 ± 1.38
PE + ENG	13.22 ± 2.51	17.74 ± 2.82	34.19	*p* < 0.001	48.34 ± 6.16	60.25 ± 8.40	24.64	*p* < 0.001	37.58 ± 0.84
PE + MI + ENG	13.05 ± 2.67	18.66 ± 1.88	42.99	*p* < 0.001	48.17 ± 6.25	64.72 ± 9.71	34.36	*p* < 0.001	40.91 ± 1.14

**Figure 2:**
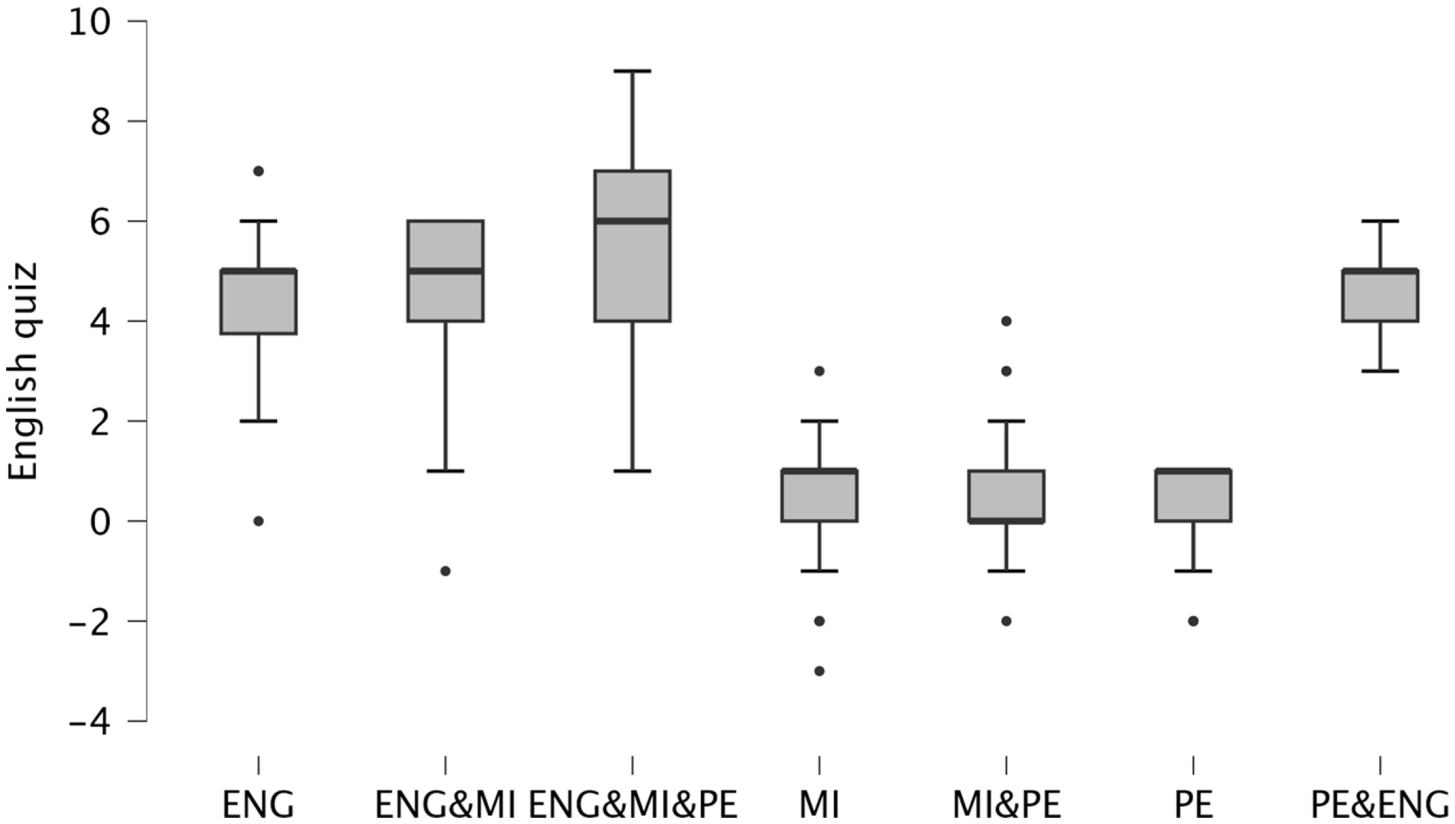
Scatterplots showing the mean differences (post–pre) in English quiz scores across the different conditions and classes. ENG, English; PE, physical education; MI: motor imagery.

All class conditions showed a significant increase in English quiz scores from pre- to post-session (all *p* < 0.001). At pre-session, there were no significant differences in English quiz scores between any of the class conditions (all pairwise comparisons *p* > 0.05). At post-session, the PE (physical education) + MI (motor imagery) + ENG (English) condition showed significantly higher post-session quiz scores compared to all other conditions (e.g., vs. ENG: mean difference = 5.609, *p* < 0.001; vs. MI: mean difference = 4.967, *p* < 0.001; vs. PE: mean difference = 4.848, *p* < 0.001). The ENG + MI condition also resulted in significantly higher post-session scores compared to MI (mean difference = 4.091, *p* < 0.001) and PE alone (mean difference = 3.972, *p* < 0.001). Furthermore, the ENG condition showed significant differences compared to MI (mean difference = 3.652, *p* < 0.001) and PE (mean difference = 3.533, *p* < 0.001), and the PE + ENG condition showed significant differences compared to MI (mean difference = 3.880, *p* < 0.001) and PE (mean difference = 3.758, *p* < 0.001). Figure 2 presents the mean difference (post–pre) in English quiz scores across the different conditions and classes.

**Figure 3:**
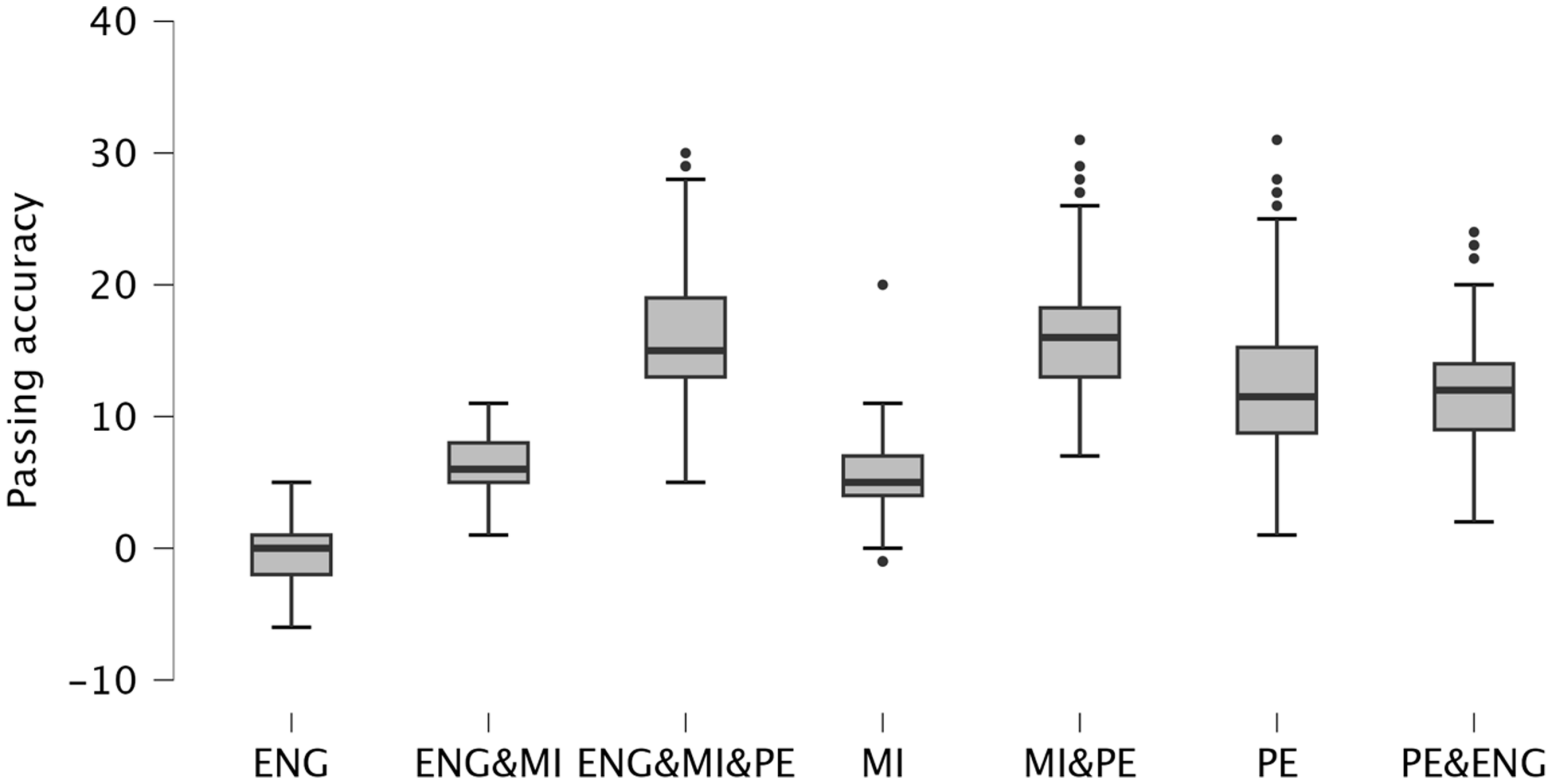
Scatterplots showing the mean differences (post–pre) in passing accuracy scores across the different conditions and classes. ENG, English; PE, physical education; MI, motor imagery.

Most class conditions showed a significant increase in passing accuracy scores from pre- to post-session (all *p* < 0.001), with the exception of the ENG class, which did not show a significant change (mean difference = 0.196, *p* = 0.371). At pre-session, there were no significant differences in passing accuracy scores between any of the class conditions (all pairwise comparisons *p* > 0.05). At post-session the PE + MI + ENG class showed significantly higher post-session passing accuracy scores compared to all other conditions (e.g., vs. ENG: mean difference = 16.511, *p* < 0.001; vs. MI: mean difference = 12.467, *p* < 0.001; vs. PE: mean difference = 3.972, *p* < 0.001). The MI + PE condition also resulted in significantly higher post-session scores compared to ENG (mean difference = 16.228, *p* < 0.001), MI (mean difference = 12.184, *p* < 0.001), and ENG + MI (mean difference = 10.185, *p* < 0.001). Furthermore, the PE condition showed significantly higher post-session scores compared to ENG (mean difference = 12.609, *p* < 0.001) and MI (mean difference = 7.337, *p* < 0.001). Figure 3 presents the mean difference (post–pre) in passing accuracy scores across the different conditions and classes.

**Figure 4:**
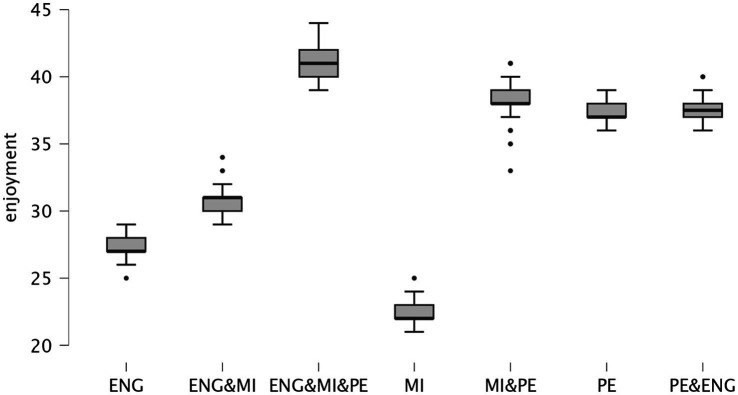
Scatterplots showing the enjoyment levels across the different conditions and classes. ENG, English; PE, physical education; MI, motor imagery.

The PE + MI + ENG condition showed significantly higher enjoyment scores compared to all other conditions (e.g., vs. ENG: mean difference = 13.500, p < 0.001; vs. MI: mean difference = 18.533, *p* < 0.001; vs. PE: mean difference = 3.478, *p* < 0.001). The MI + PE condition also resulted in significantly higher enjoyment scores compared to ENG (mean difference = 10.989, *p* < 0.001) and MI (mean difference = 16.022, *p* < 0.001). Furthermore, the PE condition showed significantly higher enjoyment scores compared to ENG (mean difference = 10.022, *p* < 0.001) and MI (mean difference = 15.054, *p* < 0.001). Conversely, the MI condition consistently showed significantly lower enjoyment scores compared to all other conditions (e.g., vs. ENG: mean difference = −5.033, *p* < 0.001; vs. PE: mean difference = −15.054, *p* < 0.001). Figure 4 presents the scatterplots of enjoyment across the different conditions and classes.

Table 11 presents the mean differences and associated *p*-values from the post-hoc pairwise comparisons between the different classes for English quiz scores (post-session), passing accuracy scores (post-session), and enjoyment scores (post-session). All p-values are adjusted for multiple comparisons using Bonferroni.

**Table 11:** Mean differences and associated *p*-values from the post-hoc pairwise comparisons between the different classes for English quiz scores (post-session), passing accuracy scores (post-session), and enjoyment scores (post-session).

Comparison (I vs. J)	English quiz post (Mean diff; *p*-value)	Passing accuracy post (Mean diff; *p*-value)	Enjoyment post (Mean diff; *p*-value)
ENG vs. MI	3.65; <0.001	−5.27; <0.001	5.03; <0.001
ENG vs. PE	3.53; <0.001	−12.61; <0.001	−10.02; <0.001
ENG vs. ENG + MI	−1.09; <0.001	−6.33; <0.001	−3.12; <0.001
ENG vs. MI + PE	−1.31; <0.001	−16.29; <0.001	−10.99; <0.001
ENG vs. PE + ENG	−1.05; <0.001	−11.91; <0.001	−10.16; <0.001
ENG vs. PE + MI + ENG	−1.97; <0.001	−16.54; <0.001	−13.50; <0.001
MI vs. PE	−0.12; 1.000	−7.34; <0.001	−15.05; <0.001
MI vs. ENG + MI	−4.74; <0.001	−1.05; <0.001	−8.15; <0.001
MI vs. MI + PE	−4.97; <0.001	−11.02; <0.001	−16.02; <0.001
MI vs. PE + ENG	−4.71; <0.001	−6.64; <0.001	−15.20; <0.001
MI vs. PE + MI + ENG	−5.62; <0.001	−11.27; <0.001	−18.53; <0.001
PE vs. ENG + MI	−4.62; <0.001	6.28; <0.001	6.90; <0.001
PE vs. MI + PE	−4.85; <0.001	−3.68; <0.001	−0.97; 1.000
PE vs. PE + ENG	−4.59; <0.001	0.70; 1.000	−0.14; 1.000
PE vs. PE + MI + ENG	−5.50; <0.001	−3.93; <0.001	−3.48; <0.001
ENG + MI vs. MI + PE	−0.22; 1.000	−9.97; <0.001	−7.87; <0.001
ENG + MI vs. PE + ENG	0.04; 1.000	−5.59; <0.001	−7.04; <0.001
ENG + MI vs. PE + MI + ENG	−0.88; <0.001	−10.22; <0.001	−10.38; <0.001
MI + PE vs. PE + ENG	0.26; 1.000	4.38; <0.001	0.83; <0.001
MI + PE vs. PE + MI + ENG	−0.65; <0.001	−0.25; 1.000	−2.51; <0.001
PE + ENG vs. PE + MI + ENG	−0.91; <0.001	−4.63; <0.001	−3.34; <0.001

ENG, English; MI, Motor Imagery; PE, physical education.

To examine the potential effects of the implemented sequencing orders (1, 2, and 3, as detailed in the Methods section), a one-way ANOVA was conducted for each condition. Table 12 presents the statistical results from comparisons between order sequences. No significant differences were found in order sequencing for the English quiz or enjoyment levels across any of the conditions (*p* > 0.05). However, sequencing order significantly affected passing accuracy in the ENG condition (order 2 had significantly lower passing accuracy than order 3, *p* = 0.004) and the ENG&MI condition (order 1 had significantly lower passing accuracy than order 3, *p* = 0.006). No other significant differences in passing accuracy were observed across the remaining conditions (*p* > 0.05).

**Table 12:** Comparison of sequencing order effects on English quiz performance, passing accuracy, and enjoyment levels.

	Order 1 (*n* = 31)	Order 2 (*n* = 31)	Order 3 (*n* = 30)	ANOVA	Significant pairwise comparisons
English quiz (mean difference post-pre)					
ENG	4.23 ± 1.23	4.42 ± 1.20	4.23 ± 0.97	*p* = 0.754; η^2^ = 0.006	
MI	0.35 ± 1.30	0.77 ± 1.02	0.80 ± 0.89	*p* = 0.203; η^2^ = 0.035	
PE	0.35 ± 1.14	0.74 ± 0.68	0.37 ± 0.85	*p* = 0.172; η^2^ = 0.039	
ENG & MI	4.74 ± 1.67	4.61 ± 2.01	4.90 ± 1.37	*p* = 0.806; η^2^ = 0.005	
MI & PE	0.77 ± 1.02	0.26 ± 0.86	0.33 ± 0.80	*p* = 0.056; η^2^ = 0.063	
PE&ENG	4.32 ± 0.94	4.68 ± 1.04	4.57 ± 0.86	*p* = 0.330; η^2^ = 0.025	
PE + MI + ENG	5.84 ± 2.20	5.00 ± 2.03	6.00 ± 1.11	*p* = 0.082; η^2^ = 0.055	
Passing accuracy (mean difference post-pre)					
ENG	−0.39 ± 1.87	−0.94 ± 1.88	0.77 ± 2.19	*p* = 0.004*; η^2^ = 0.116	Order 2 < 3; p = 0.004
MI	5.64 ± 3.87	4.64 ± 1.87	5.53 ± 2.34	*p* = 0.319; η^2^ = 0.025	
PE	12.84 ± 6.50	12.51 ± 5.78	12.47 ± 5.30	*p* = 0.964; η^2^ = 0.001	
ENG & MI	5.45 ± 2.19	6.52 ± 1.84	7.03 ± 1.75	*p* = 0.007*; η^2^ = 0.106	Order 1 < 3; p = 0.006
MI & PE	14.71 ± 3.42	16.74 ± 6.03	17.47 ± 4.97	*p* = 0.081; η^2^ = 0.055	
PE & ENG	12.52 ± 4.34	11.48 ± 3.81	11.73 ± 4.35	*p* = 0.599; η^2^ = 0.011	
PE + MI + ENG	17.55 ± 5.78	16.74 ± 5.30	15.30 ± 5.51	*p* = 0.280; η^2^ = 0.028	
Enjoyment					
ENG	27.65 ± 0.80	27.19 ± 0.91	27.40 ± 0.93	*p* = 0.136; η^2^ = 0.044	
MI	22.39 ± 0.76	22.55 ± 0.77	22.20 ± 0.89	*p* = 0.246; η^2^ = 0.031	
PE	37.58 ± 0.89	37.48 ± 0.93	37.23 ± 0.82	*p* = 0.287; η^2^ = 0.028	
ENG & MI	30.65 ± 1.14	30.55 ± 0.93	30.40 ± 0.86	*p* = 0.620; η^2^ = 0.011	
MI & PE	38.23 ± 1.06	38.42 ± 1.93	38.57 ± 0.97	*p* = 0.632; η^2^ = 0.010	
PE & ENG	37.48 ± 0.81	37.58 ± 0.81	37.67 ± 0.92	*p* = 0.702; η^2^ = 0.008	
PE + MI + ENG	40.74 ± 1.29	40.84 ± 1.00	41.17 ± 1.09	*p* = 0.315; η^2^ = 0.026	

*Significantly different.

## 4 Discussion

The present study investigated the immediate effects of various class conditions (English, MI, PE, and their combinations) on English quiz scores, passing accuracy, and session enjoyment. For English quiz scores, improvement from pre- to post-session was observed across all conditions, coupled with significant differences between class types and an interaction indicating that the magnitude of improvement varied considerably depending on the specific class combination, with combined modalities generally yielding greater positive impact. Similarly, passing accuracy showed significant overall improvement over time and significant differences between conditions. While most class types led to enhanced passing accuracy, the English-only condition did not show a significant change, whereas conditions incorporating PE and MI, particularly in combination, exhibited the most substantial positive effects. Finally, post-session enjoyment varied significantly across class conditions, with combined approaches, especially those integrating PE and MI, reporting higher levels of enjoyment, contrasting with the relatively lower enjoyment reported for the MI-only condition.

Conditions combining English instruction with other subjects, particularly PE + MI + ENG and ENG + MI, showed the most substantial positive impacts in post-session quiz scores. This finding aligns with the growing body of literature on multimodal learning and integrated curricula, which posits that engaging multiple sensory and cognitive pathways can enhance learning outcomes (Faella et al., 2025). Beyond traditional cognitive benefits such as increased neurotrophic factors and improved synaptic plasticity associated with exercise (Vaynman et al., 2003), these outcomes may be partly explained by principles of embodied cognition (Atkinson, 2010). In this context, PE may provide sensorimotor input, potentially grounding abstract linguistic concepts in concrete bodily experiences (Mazzuca et al., 2021). For instance, learning action verbs while performing the actions can create stronger, more accessible neural representations (Bedny and Caramazza, 2011). Similarly, MI, while a covert process, involves the mental simulation of actions (Decety, 1996), activating neural networks similar to those engaged during actual movement (Sharma and Baron, 2013). This fact could contribute to a more embodied understanding of language, particularly if the language content relates to actions, movements, or spatial relationships (Willems and Casasanto, 2011). However, the results may also be related to specific curriculum designs, and future research is needed to understand how pedagogical approaches and curriculum design may interact.

Regarding passing accuracy, the results showed a significant overall improvement from pre- to post-session, a significant effect of class condition, and an interaction between time and class. This interaction highlights that while most class conditions led to increases in passing accuracy, the magnitude of improvement varied. The English-only condition did not yield a significant change in passing accuracy, which is justified by the absence of any instruction or motor skill engagement. In contrast, conditions incorporating PE and MI, particularly the MI + PE and PE + MI + ENG combinations, exhibited the most substantial improvements. These findings are consistent with research showing the efficacy of skill training practice in basketball motor skill acquisition (Fikri et al., 2023). The improvements seen in classes incorporating MI are also consistent with a systematic review which revealed positive impacts on developing sport-specific motor skills (Lindsay et al., 2023). Studies have showed that mental practice can lead to performance improvements comparable to physical practice, especially when combined as observed in a review about the topic (Behrendt et al., 2021). This dual-modality approach may enhance learning by engaging both direct sensorimotor pathways and cognitive simulation (Guillot et al., 2024). This is particularly relevant for adolescents, who may perceive MI as abstract unless it is supported by concrete scaffolding, highlighting the importance of integrating tangible or guided experiences to make MI more accessible and effective.

Consistent with the pattern observed in the other measures, conditions incorporating multiple approaches, particularly PE + MI + ENG and MI + PE, garnered the highest enjoyment ratings. Conversely, the MI-only condition was associated with significantly lower enjoyment compared to all other groups. These findings align with the need for increasing intrinsic motivation and engagement in educational and physical activity contexts (Adank et al., 2024). PE, by its very nature, often involves dynamic, interactive, and varied movements namely in those using games, which can be inherently enjoyable (Mo et al., 2024). Similarly, multimodal learning approaches, which integrate various sensory inputs and activity types, are generally found to increase student engagement and motivation (Xiao et al., 2022). The lower enjoyment in the MI-only condition, despite its showed benefits, might be attributed to its less overtly interactive nature. While mentally stimulating, the lack of physical exertion, social interaction, and external stimuli typically present in PE or combined classes could lead to a diminished sense of enjoyment for some participants. This highlights a potential trade-off between the focused, internal cognitive work of MI and the broader, more intrinsically rewarding experience offered by physically active and multimodal learning environments.

Despite these findings, the present study has several limitations. One limitation is the absence of a washout period, which may have inflated cumulative learning effects. Future research should incorporate washout periods to minimize the risk of carryover effects and ensure that comparisons between conditions are not compromised. Another limitation is that including all seven conditions, which cover every possible combination of PE, MI, and ENG, made it difficult to implement all 5,040 possible randomization orders. Consequently, only three sequences were used, which do not cover all possible permutations. This may introduce some limitations related to the specific sequences implemented; however, this approach is justified by the practical constraints of conducting the study within a real educational setting. Future research should also incorporate longitudinal designs (e.g., 6 weeks with retention tests) to investigate the sustained impact of these multimodal approaches, as the current study reflects only short-term skill development across sessions. Moreover, a non-crossover control group (following the standard curriculum) could be included in future research. Additionally, mediation analyses (e.g., examining attention or motivation) should be included to explore potential driving effects in future studies. Additionally, the study relied on self-reported enjoyment, which could be subject to social desirability bias. Due to the nature of the study design, it is important to recognize that the novelty of the methods may influence enjoyment. Only in longitudinal studies would it be possible to mitigate such potential bias. Future studies could also delve deeper into the specific mechanisms underlying the observed enjoyment differences, perhaps through qualitative methods to capture participants’ subjective experiences. Furthermore, concurrent activation of motor (PE) and semantic (MI) networks may enhance vocabulary retention, but neurophysiological validation (e.g., electroencephalography or functional near-infrared spectroscopy) is warranted in future studies. Additionally, future research should examine the effects on abstract vocabulary, not just action vocabulary, as well as testing the effects in different age groups.

Practically, these findings suggest that educators and coaches should consider integrating diverse modalities into their teaching practices to enhance student engagement and enjoyment. Incorporating brief physical activity breaks or guided MI sessions into traditional language classes could not only boost learning outcomes but also foster a more positive and motivating learning environment. For example, educators might implement PE-based games (e.g., ‘Simon Says’ for action verbs) and scripted MI audio guides (approximately 5 min in duration) to facilitate feasible classroom integration.

## 5 Conclusion

In conclusion, this provides evidence that integrating diverse disciplines—English instruction, MI, and PE—can significantly influence language retention (English quiz scores) and motor (passing accuracy) learning outcomes, as well as affective responses (enjoyment). The superior performance and higher enjoyment reported in combined conditions suggest the potential synergistic benefits of multimodal learning approaches. These findings advocate for a more holistic and integrated approach to education and skill development, suggesting that combining traditional academic instruction with physical and mental training can lead to more effective, engaging, and enjoyable learning experiences. Future research should build upon these immediate findings to explore long-term retention and the generalizability of these benefits across different learning contexts and populations.

## Data Availability

The raw data supporting the conclusions of this article will be made available by the authors, without undue reservation.
